# How does blood regulate cerebral temperatures during hypothermia?

**DOI:** 10.1038/s41598-018-26063-7

**Published:** 2018-05-18

**Authors:** Stephen Blowers, Ian Marshall, Michael Thrippleton, Peter Andrews, Bridget Harris, Iain Bethune, Prashant Valluri

**Affiliations:** 1 Institute of Multiscale Thermofluids, School of Engineering, University of Edinburgh, Edinburgh, UK; 2 Centre for Clinical Brain Sciences, College of Medicine and Veterinary Medicine, University of Edinburgh, Edinburgh, UK; 3 Anaesthesia, Critical Care and Pain Medicine, University of Edinburgh, Edinburgh, UK; 4 STFC Hartree Centre, Daresbury Laboratory, Warrington, UK

## Abstract

Macro-modeling of cerebral blood flow can help determine the impact of thermal intervention during instances of head trauma to mitigate tissue damage. This work presents a bioheat model using a 3D fluid-porous domain coupled with intersecting 1D arterial and venous vessel trees. This combined vascular porous (VaPor) model resolves both cerebral blood flow and energy equations, including heat generated by metabolism, using vasculature extracted from MRI data and is extended using a tree generation algorithm. Counter-current flows are expected to increase thermal transfer within the brain and are enforced using either the vascular structure or flow reversal, represented by a flow reversal constant, *C*_*R*_. These methods exhibit larger average brain cooling (from 0.56 °*C* ± <0.01 °*C* to 0.58 °*C* ± <0.01 °*C*) compared with previous models (0.39 °*C*) when scalp temperature is reduced. An greater reduction in core brain temperature is observed (from 0.29 °*C* ± <0.01 °*C* to 0.45 °*C* ± <0.01 °*C*) compared to previous models (0.11 °*C*) due to the inclusion of counter-current cooling effects. The VaPor model also predicts that a hypothermic average temperature (<36 °*C*) can be reached in core regions of neonatal models using scalp cooling alone.

## Introduction

It is widely accepted in the medical community that a reduction of brain temperature after cardiac arrest^1^ and brain trauma^2^ is beneficial to the outcome of the patient. The target temperature for hypothermia is defined as a core temperature <36 °*C*^3^ but even a fraction of a degree Celsius change could be clinically relevant^4^. Unfortunately, direct measurement of brain temperature is generally difficult and invasive, although Magnetic Resonance Imaging (MRI) holds some promise for future studies^5^. Potential temperature management interventions are difficult to develop and then verify without direct observation of deep brain temperatures. Therefore, cerebral temperature predictions are made with mathematical models to investigate possible efficacy prior to initiating clinical trials. This paper proposes a novel three dimensional brain temperature model incorporating arterial and venous vasculature coupled with porous tissue. This allows the prediction of both flow and temperature profiles in the entire brain tissue.

Modelling of brain tissue heat transfer and cerebral cooling has mostly relied on variants of Pennes’ Bioheat Equation (PBE)^6^ given by Equation 1. This equation models the metabolic heat generation and conductive heat transfer of tissue coupled with a perfusion term which represents the heat transfer to and from blood delivered to the tissue:

1 $$\rho c\frac{\partial T}{\partial t}=K{\nabla }^{2}T-{c}_{b}\omega (T-{T}_{a})+{Q}_{gen}$$

where *ρ* is density (*kg*/*m*^3^), *c* is specific heat capacity (*J*/*kg*/°*C*), *T* is temperature (°*C*), *t* is time (*s*), *K* is the conductive heat transfer coefficient (*W*/*m*/°*C*), and *Q*_*gen*_ represents metabolic heat generation of tissue (*W*/*m*^3^). The term, *ω*, is a perfusion term that represents the flow rate of blood per volume of tissue (*kg*/*m*^3^/*s*, however, usually quoted in units of *ml*/100*g*/*min*). The subscript, *b*, represents a property of blood whereas physical values without subscripts denote properties of tissue. The term *T*_*a*_ signifies arterial blood temperature (°*C*).

These models based on PBE make many simplifications, primarily assuming blood flow is dictated by a perfusion term with no directional parameter and simplified geometries to facilitate computation^7–9^. They conclude that the effect of surface temperature on core brain tissue is negligible because of high cerebral blood perfusion. This was dubbed the so-called ‘temperature shielding effect’ of blood flow^10^ and showed that a reduction in tissue temperature from surface cooling only occurred in the outer 1–2 *cm* of the brain tissue. This model agreed well with vertical temperature measurements and perfusion predictions in rat brains^10,11^.

Criticisms of PBE highlight the lack of flow direction and transport effects of blood^12–14^. More recently, the bioheat models have benefited from the inclusion of vasculature. The DiVa (Discrete Vasculature) Model includes 1D vessel segments by establishing convective heat transfer between vessels and tissues through the use of bucket nodes, which act as heat sinks in the tissue^15^. A perfusion zone is established at segment ends and branch intersections to model the blood distribution in capillaries throughout the vessel network and to balance the mass transfer^16^. This perfusion zone operates the same as the perfusion term in Equation 1 whereby the local transport effects of blood are not present, and important heat transfer effects could be omitted. The model was validated against temperatures measured from a bovine tongue^17^, and then used to investigate the potential effect of cooling caps on neonatal heads^18^. The results produced were in good agreement with the shielding effect theory, however, are not representative of clinical measurements by Harris *et al*.^19^ that indicate core temperatures can be affected by surface cooling. The use of this perfusion zone is derived from the idea that small vessels, below 0.2 *mm* in diameter, rapidly reach equilibrium with the surrounding tissue and become ‘thermally insignificant’^20^. However, this assumption then ignores the directional flow and counter-current effects associated with these small vessels. The current work presents a model that includes directional flow at all scales of cerebral blood circulation and demonstrates the effect this has on potential treatments, such as scalp cooling. This is achieved by embedding 1-D vasculature in a 3-D porous media domain and thus is named the Vascular Porous (VaPor) method.

## Methods

### VaPor Model Overview

**Figure 1:**
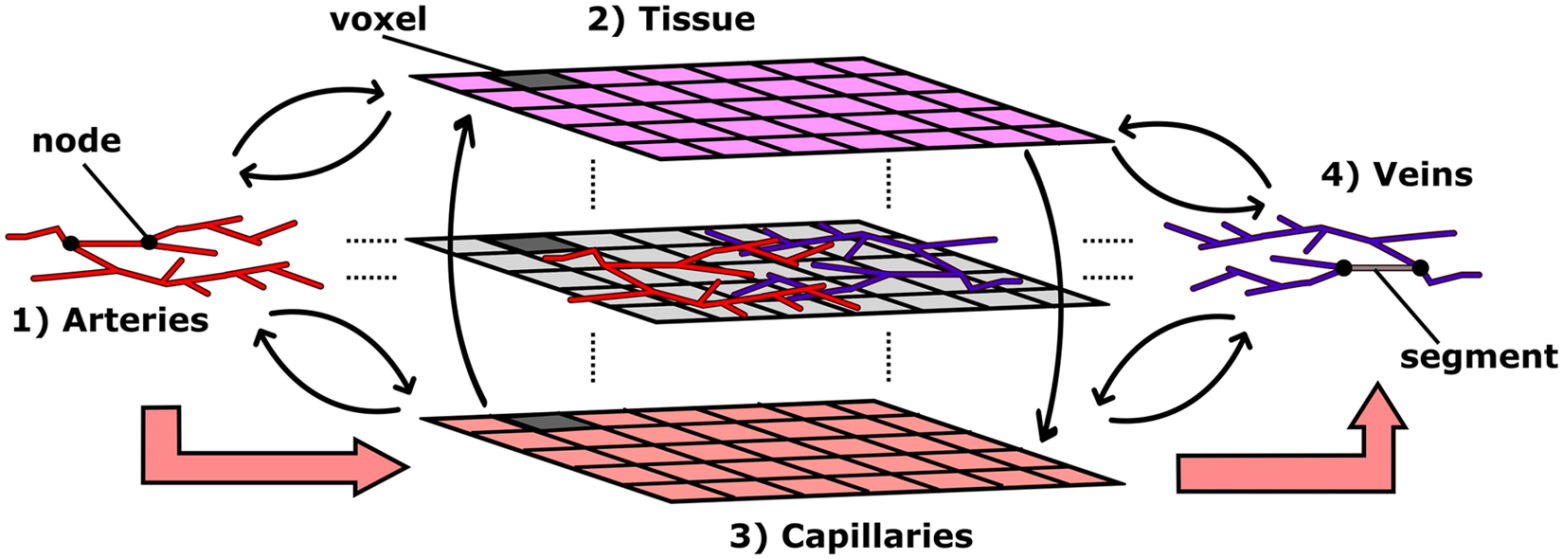
A depiction of the domain structures that the VaPor model uses. The arteries and veins which are 1-Dimensional vessel domains are embedded in a 3-Dimensional domain that is comprised of tissue and capillaries. Heat transfer occurs between all domains except the arteries and veins, as depicted by the thin black arrows. Flow travels from the arteries, to the capillaries, and then to the veins as depicted by the thick red arrows. Additionally, for clarification, the terms ‘voxel’, ‘node’, and ‘segment’ are illustrated here. A ‘voxel’ is a volumetric element of the 3D domains. The shaded examples here all occupy the same space with each domain occupying a certain fraction of the total volume. The ‘nodes’ are points defined by the vessel data sets whereas the ‘segments’ are defined by the connections between ‘nodes’. Whilst most information is stored at the ‘nodes’, the interaction between domains occurs through the ‘segments’.

The concept of the model is to simulate blood flow through both vessels and tissue and subsequently calculate the energy balance of the system to determine cerebral temperatures. This allows the prediction of tissue temperatures while avoiding the stringent assumptions made by continuum models regarding blood transportation within the perfusion term of Equation 1. This bears some similarity to the DiVa model^15^ only in that the large arterial and venous vessels are modelled as 1D line segments embedded inside a 3D domain. The structure of the model is displayed in Fig. 1. However, unlike the DiVa model that assigns a single perfusion term to the tissue as in Equation 1, the VaPor model solves the 3D transient flow and energy transport through the 3D tissue domain as if it were a porous tissue. The blood is then retrieved into the venous vessels and removed at the outlets of the model.

### Temperature Model

This model solves temperature within four main domains: the first, being the temperature within the artery line segments, the second being the tissue temperatures within the porous region, the third being the blood temperatures within the porous region, and the fourth being the venous line segments. For the purposes of the explanation these are designated the subscripts 1, 2, 3, and 4, respectively. The equations for temperature in each domain are given as:

Arterial:

2 $${V}_{1}{\rho }_{b}{c}_{b}\frac{\partial {T}_{1}}{\partial t}={V}_{1}{K}_{b}({\nabla }^{2}{T}_{1})-{V}_{1}{\rho }_{b}{c}_{b}{U}_{1}(\nabla {T}_{1})+{\beta }_{1\leftrightarrow 2}({T}_{2}-{T}_{1})+{\beta }_{1\leftrightarrow 3}({T}_{3}-{T}_{1})$$

Tissue:

3 $${V}_{2}{\varepsilon }_{2}\rho c\frac{\partial {T}_{2}}{\partial t}={V}_{2}{\varepsilon }_{2}K({\nabla }^{2}{T}_{2})-{\beta }_{1\leftrightarrow 2}({T}_{2}-{T}_{1})+{\beta }_{2\leftrightarrow 3}({T}_{3}-{T}_{2})+{\beta }_{2\leftrightarrow 4}({T}_{4}-{T}_{2})+{V}_{2}{Q}_{gen}$$

Blood in Tissue:

4 $$\begin{array}{rcl}{V}_{3}{\varepsilon }_{3}{\rho }_{b}{c}_{b}\frac{\partial {T}_{3}}{\partial t} & = & {V}_{3}{\varepsilon }_{3}{K}_{b}({\nabla }^{2}{T}_{3})-{V}_{3}{\rho }_{b}{c}_{b}{U}_{3}(\nabla {T}_{3})-{\beta }_{1\leftrightarrow 3}({T}_{3}-{T}_{1})\\  &  & -{\beta }_{2\leftrightarrow 3}({T}_{3}-{T}_{2})+{\beta }_{3\leftrightarrow 4}({T}_{4}-{T}_{3})+{c}_{b}{\dot{M}}_{1\to 3}({T}_{3}-{T}_{1})\end{array}$$

Venous:

5 $$\begin{array}{rcl}{V}_{4}{\rho }_{b}{c}_{b}\frac{\partial {T}_{4}}{\partial t} & = & {V}_{4}{K}_{b}({\nabla }^{2}{T}_{4})-{V}_{4}{\rho }_{b}{c}_{b}{U}_{4}(\nabla {T}_{4})-{\beta }_{2\leftrightarrow 4}({T}_{4}-{T}_{2})-{\beta }_{3\leftrightarrow 4}({T}_{4}-{T}_{3})\\  &  & +{c}_{b}\,{\dot{M}}_{3\to 4}({T}_{4}-{T}_{3})\end{array}$$

Here, *V* denotes the volume (i.e. segment or voxel) of domain concerned (*m*^3^), *U* is the velocity of blood (*m*/*s*), *ε*_2_ is the volume fraction of tissue within the porous domain, and *ε*_3_ is the volume fraction of blood (with *ε*_2_ = 1 − *ε*_3_). The term ${\dot{M}}_{1\to 3}$ and ${\dot{M}}_{3\to 4}$ represent mass transport between the indicated domains (*kg*/*s*). The term *Q*_*gen*_ represents the metabolic heat generation of the tissue (*W*/*m*^3^).

The value *β* represents the inter-domain heat transfer between the indicated domains (*W*/°*C*). At the capillary level, heat transfer between blood and tissue is expected to rapidly reach equilibrium^20^ so the coefficient for heat transfer in the 3D domain, *β*_2↔3_, was set to a high-value representing infinity. This means that at the capillary level, *T*_2_ = *T*_3_. The inter-domain heat transfer between 3D domains and vessel domains is derived from Newtonian heat transfer for laminar flow as in the DiVa Model^15^:

6 $${\beta }_{1\leftrightarrow 2}={\varepsilon }_{2}\,Nu\,\frac{{K}_{b}}{{D}_{1}}\frac{\pi {D}_{1}{L}_{1}}{N}$$

7 $${\beta }_{2\leftrightarrow 4}={\varepsilon }_{2}\,Nu\,\frac{{K}_{b}}{{D}_{4}}\frac{\pi {D}_{4}{L}_{4}}{N}$$

where *Nu* is the Nusselt number, *D* is the diameter of the vessel segment (*m*), and *L* is the length of the vessel segment (*m*). For simplicity, the inter-domain heat transfer is divided equally between the voxels intersected by a vessel segment, the number of voxels being denoted by *N*. The blood flows and diameters in all vessels in question are small enough that only laminar flow is present. For fully developed laminar flow, the Nusselt number can be approximated to a constant, typically *Nu* ≈ 4^21^. The equations for *β*_1↔3_ and *β*_3↔4_ are identical except for replacing *ε*_2_ with *ε*_3_.

### Flow Model

Figure 1 shows how the vessel domains interact with the porous domains. The 1D line segments that make up the domains 1 and 4 interact with voxels in domains 2 and 3 only where they intersect one another. Even though the diameter of the vessel may occupy multiple voxels in the 3D domain, for simplicity only the intersected voxels are considered adjacent. Additionally, heat transfer and mass transfer parameters are divided equally among the intersecting voxels regardless of length of intersection by the corresponding vessel segment.

As the inlets and outlets of the vascular trees are defined, velocities required for the advection terms in Equations 2 and 5, can be caluclated using the conservation of mass:

Arteries:

8 $$\sum {\dot{F}}_{1}=-\,{\dot{M}}_{1\leftrightarrow 3}$$

Veins:

9 $$\sum {\dot{F}}_{4}={\dot{M}}_{3\leftrightarrow 4}$$

where $\dot{F}$ is the mass flow rate within the vessel segments (*kg*/*s*) and the summation i*s* over all connections for each node. The mass flow rate can be converted to velocities by the relationship:

10 $$U=\frac{4\dot{F}}{{\rho }_{b}\pi {D}^{2}}$$

The vessel diameters, *D*, are designated by the flow rate through them using a relationship derived from the data of blood velocities within blood vessels^22^ as shown in Equation 11:

11 $$D=0.0332{\dot{F}}^{0.3703}$$

This calculates diameters of 4.9 *mm* in the internal carotid arteries which lie in the expected range^23^. Diameters at branch terminations at the furthest generation do not fall beneath 17 *μm* which lies in the expected range for metarterioles^24^. It is assumed that vessel volumes are small enough that they do not affect the volumes of voxels that they intersect $({V}_{1}+{V}_{4}\ll {V}_{2}+{V}_{3})$.

The inter-domain mass transfer terms, $\dot{M}$, dictate the transfer of blood between indicated domains in the subscript. The values are assigned arbitrarily to the vessel segments that are located at branch terminations based on their length ($\dot{M}\propto {L}_{BT}$) where *L*_*BT*_ is the length of a vessel segment that is a branch termination. All other segments have no inter-domain mass transfer $(\dot{M}=0)$. Initial studies sought to couple $\dot{M}$ with pressure gradient, as done with previous models^25^; however, this resulted in many regions remaining poorly perfused with flow tending to ‘short-circuit’ between arterial and venous vessel trees. As with the inter-domain heat transfer term, *β*, the values for $\dot{M}$ are distributed equally between the intersecting voxels in domain 3. Assigning inter-domain mass transfer terms allows the three domains to be solved independently.

For the velocities in Equation 3, the vascular resistance method^25^ is used:

12 $${U}_{3}=\frac{{G}_{3}{\rm{\Delta }}{P}_{3}}{{\rho }_{b}{l}^{2}}$$

13 $$\sum {G}_{3}{\rm{\Delta }}{P}_{3}={\dot{M}}_{1\leftrightarrow 3}-{\dot{M}}_{3\leftrightarrow 4}$$

where *l* is the length of a voxel edge (*m*), *P* is pressure (*Pa*), and *G* represents the vascular conductance (*m* ⋅ *s*). The summation occurs over each neighbouring voxel. The value for *G* is given by a partial derivation of the Carman-Kozeny Equation:

14 $${G}_{3}=\frac{{\rho }_{b}{\varepsilon }_{3}l\pi {{D}_{c}}^{2}}{32{\mu }_{b}\tau }$$

where *μ*_*b*_ is the viscosity of blood (*Pa* ⋅ *s*), *D*_*c*_ is the capillary diameter which is set at 10 *μm*, and *τ* is the tortuosity of capillary blood vessels which is set to 1.6^26^. Variations of the values of *D*_*c*_ and *τ* between 1–50 *μm* and 1–5 respectively had negilgable effect on temperature results (<0.001 °*C*).

### Flow Reversal

To include counter-current effects of tortuous vessels in the capillary region, Equation 4 can be replaced with:

Blood in Tissue:

15 $$\begin{array}{rcl}{V}_{3}{\varepsilon }_{3}{\rho }_{b}{c}_{b}\frac{\partial {T}_{3}}{\partial t} & = & {V}_{3}{\varepsilon }_{3}{K}_{b}({\nabla }^{2}{T}_{3})-{V}_{3}{\rho }_{b}{c}_{b}{U}_{3A}(\nabla {T}_{3})+{V}_{3}{\rho }_{b}{c}_{b}{U}_{3B}(\nabla {T}_{3})\\  &  & -{\beta }_{1\leftrightarrow 3}({T}_{3}-{T}_{1})-{\beta }_{2\leftrightarrow 3}({T}_{3}-{T}_{2})+{\beta }_{3\leftrightarrow 4}({T}_{4}-{T}_{3})\\  &  & +{c}_{b}\,{\dot{M}}_{1\to 3}({T}_{3}-{T}_{1})\end{array}$$

where *U*_3*A*_ and *U*_3*B*_ represent forward and reverse vectors of the net flow direction (*m*/*s*), with *U*_3_ = *U*_3*A*_ + *U*_3*B*_. It is logical to assume that the forward flow is directly proportional to the net flow. Thus, considering *C*_*R*_ as a counter-current factor we can write:

16 $${U}_{3A}={C}_{R}{U}_{3}$$

17 $${U}_{3B}=-\,({C}_{R}-1){U}_{3}$$

The net mass balance is conserved and the counter-current effects occur with any value of |*C*_*R*_| > 1. A value of *C*_*R*_ = 1 reduces Equation 15 back to Equation 4.

### Human Head Domain Creation

**Figure 2:**
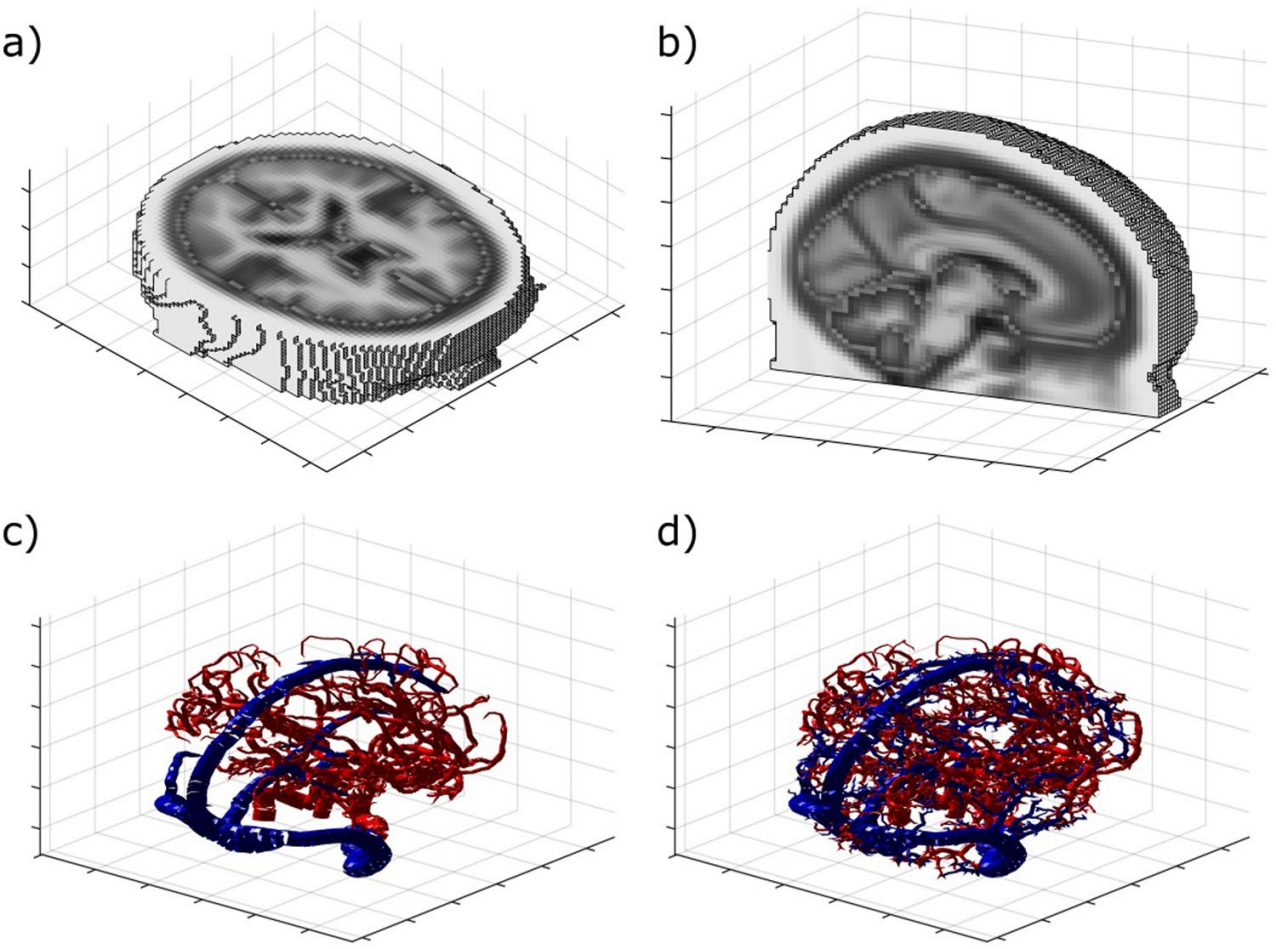
Top Row: An axial (**a**) and sagittal (**b**) slice of the head and brain probability maps used for all trials with the VaPor model. The various tissue types were assigned a shade of grey to highlight the segmentation of the domain. Bottom Row: Vessel expansion using RRT Method and diameters from flow. (**c**) Original vessel structure. (**d**) Vessel structure after 2500 iterations of RRT algorithm.

The human head domain was derived from SPM12 tissue probability maps (found at www.fil.ion.ucl.ac.uk/spm). Cross sections of the data are shown in Fig. 2a,b. The data was divided into a 60 × 72 × 60 voxel domain with an isotropic voxel length of 3 *mm*. Only voxels that were ≥50% grey and white matter were considered to be part of the brain domain (domains 2 and 3). Properties of tissue were assigned to each voxel weighted by the composition of tissue type, the values can be found in Table 1. For simplicity, the surrounding tissue outside the brain domain was assigned a perfusion term and temperatures were solved using Equation 1 and the cerebral spinal fluid (CSF) surrounding the brain is assumed to be stationary. A mesh dependency test showed that a reduction of voxel size to 2.25 *mm* led to <0.1% average difference in results.

**Table 1:** Table of physical parameters used in the VaPor model.

Physical Parameter	Value	Units	Physical Parameter	Value	Units
Arterial Blood Temperature, *T*_*a*_	37^†^	°*C*	Perfusion Rate, *ω*		
Blood Viscosity, *μ*_*b*_	3.5	*mPa*⋅*s*	Grey Matter	80^†^	*ml*/100*g*/*min*
Blood Volume Fraction, *ε*_*b*_			White Matter	20^†^	*ml*/100*g*/*min*
Grey Matter	0.0672^*^	—	CSF	0^‡^	*ml*/100*g*/*min*
White Matter	0.0405^*^	—	Bone	1.8^†^	*ml*/100*g*/*min*
Capillary Diameter	10	*μm*	Scalp	2.0^†^	*ml*/100*g*/*min*
Capillary Tortuosity	1.6	—	Specific Heat Capacity, *c*		
Density, *ρ*			Tissue		
Tissue			Grey Matter	3700^†^	*J*/*kg*/°*C*
Grey Matter	1030^†^	*kg*/*m*^3^	White Matter	3700^†^	*J*/*kg*/°*C*
White Matter	1030^†^	*kg*/*m*^3^	CSF	3800^‡^	*J*/*kg*/°*C*
CSF	1000^‡^	*kg*/*m*^3^	Bone	1590^†^	*J*/*kg*/°*C*
Bone	1520^†^	*kg*/*m*^3^	Scalp	4000^†^	*J*/*kg*/°*C*
Scalp	1000^†^	*kg*/*m*^3^	Blood	3800^†^	*J*/*kg*/°*C*
Blood	1050^†^	*kg*/*m*^3^	Thermal Conductivity, *K*		
Metabolic Heat Generation, *Q*_*gen*_			Tissue		
Grey Matter	16700^†^	*W*/*m*^3^	Grey Matter	0.49^†^	*W*/*m*/°*C*
White Matter	4175^†^	*W*/*m*^3^	White Matter	0.49^†^	*W*/*m*/°*C*
CSF	0^‡^	*W*/*m*^3^	CSF	0.5^‡^	*W*/*m*/°*C*
Bone	368.3^†^	*W*/*m*^3^	Bone	1.16^†^	*W*/*m*/°*C*
Scalp	363.4^†^	*W*/*m*^3^	Scalp	0.342^†^	*W*/*m*/°*C*
Nusselt Number, *Nu*	4	—	Blood	0.492^†^	*W*/*m*/°*C*

^†^Values taken from Neimark *et al*.^35^. ^‡^Values taken from van Leeuwen *et al*.^18^. ^*^Values calculated from cerebral blood volume values given in Larsson *et al*.^37^.

### Vessel Generation

**Figure 3:**
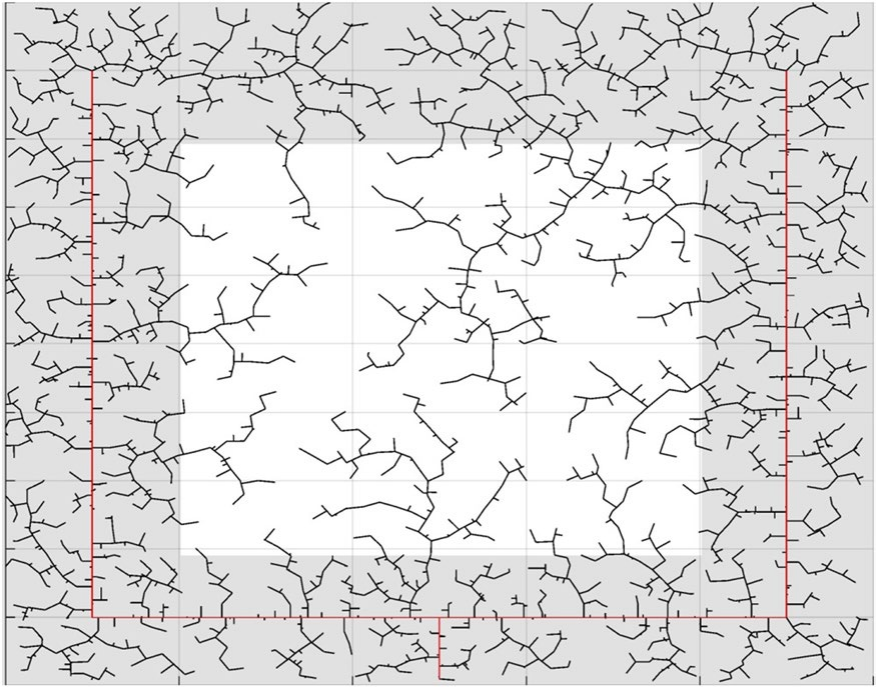
RRT generation for a 2D domain at 2,500 iterations. The red segments depict a pre-allocated vessel structure. The shaded area has 4× higher perfusion than the unshaded region and, therefore, attracts 4× the number of vessel segments.

The base arterial vessel tree (see Fig. 2c) used within the model was extracted from BraVa database (downloaded from http://cng.gmu.edu/brava)^27^ and the base venous vessel tree was extracted from a separate MR venogram. These were used without alteration in the trials that incorporated the flow reversal constant, *C*_*R*_. However, in order to include vessels invisible to the scan, the base arterial and venous vessel trees are expanded upon using a Rapidly-exploring Random Tree (RRT) algorithm^28^. The space-filling algorithm procedurally generates coordinates within the brain domain, weighted by predicted tissue perfusion values from Table 1, and then creates a vessel segment by connecting them to the existing tree by the nearest branch or node. The length of this connection is capped at an arbitrary length of 3 *mm*. In the case of joining a segment, the segment is divided into two at the point of intersection. Figure 2d shows the vessel trees after 2,500 iterations of the RRT algorithm. An example of the algorithm in 2D is shown in Fig. 3.

Inlet flow rates for the arteries were split 40%, 40%, 20% between the left and right internal carotid arteries and basilar artery respectively based on relationships in Tanaka *et al*.^29^. The outlet flow rates in the veins were split equally between the two transverse sinuses. While a pressure boundary could be used at vessel terminations in order to naturally solve for outlet flow rates, this method facilitates the calculation of vessel diameters. Adjusting the distribution of flow at the outlets to favour one side or the other has negligible effects on the results. The inlet blood flow rate was assumed to be at steady state as it has been shown that the effects of pulsatile flow are minimal within the vessel diameter ranges in question^30^. Total inlet flow rate was calculated to be 14.1 *g*/*s* from the perfusion data of grey and white matter in Table 1.

### Model Implementation

The above model was implemented as described above using a custom Matlab code. The velocities for domains 1, 3, and 4 are solved independently through matrix inversion. The temperatures for all four domains are then solved simultaneously via matrix inversion using the derived velocities.

A typical solution with a 3 *mm* voxel size and generating 200,000 vessels takes 20 mins in Matlab on a laptop with an Intel i5-3340M QuadCore processor and 8GB of RAM.

### Data availability

The data that support the findings of this study are available from the corresponding author upon reasonable request. Additionally, an open source version of the code solving the VaPor equations is available at: https://github.com/sblowers/VaPor.

## Results

### Cooling an Adult Brain

The VaPor model is tested on a human head to explore the effects of scalp cooling on the brain and how the convective effects of blood flow could have an impact. The surface temperature of the head was set to either 33.5 °*C* to represent normal scalp temperature or 10 °*C* to represent cooling the scalp. This was performed with the base vessel tree and 2,500 generated vessels with various values for the flow reversal constant. Additionally, various levels of iterations (2,500–200,000) of vessel generation with *C*_*R*_ = 1 were explored. The variation between generated vessel trees was observed by producing 10 separate trees.

**Figure 4:**
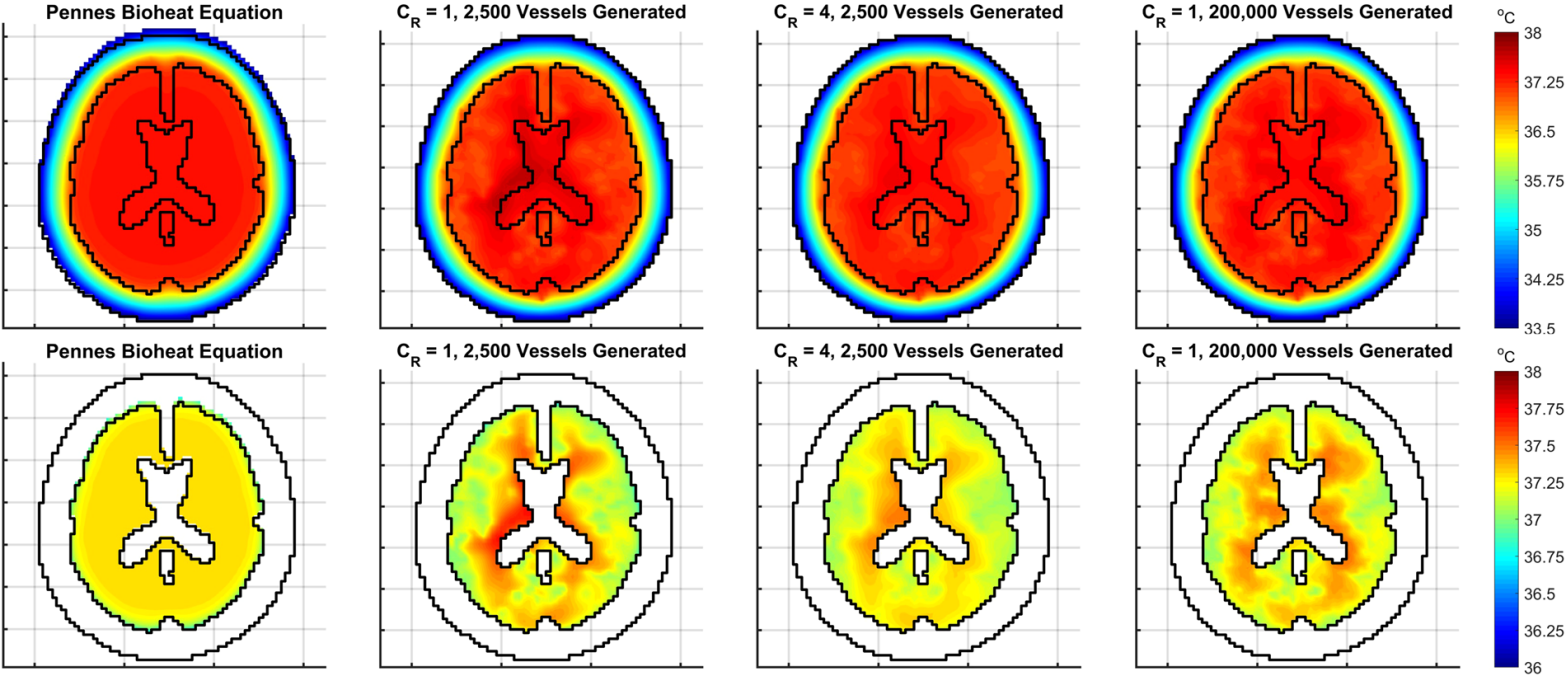
Temperature profiles for the head and brain tissue with scalp temperature set at 33.5 °*C* in an adult head. The top row shows temperatures for the whole head whereas the bottom row only shows brain temperatures to highlight the variations of temperature within the tissue.

Figure 4 shows the cerebral tissue temperature profiles with the scalp temperature set to 33.5 °*C*. Average brain temperature is consistent though each variation of the model, varying from 37.22 °*C* (±<0.01 °*C*, all errors denote the standard error across the 10 different vascular trees used) to 37.24 °*C* (±<0.01 °*C*) within the range of *C*_*R*_ = 1 to *C*_*R*_ = 7, and varying from 37.24 °*C* (±<0.01 °*C*) to 37.27 °*C* (±<0.01 °*C*) within the range of 2,500 to 200,000 generated vessels. The average temperature for the trial with PBE is 37.24 °*C*. With only the base vessel trees and *C*_*R*_ = 1, some regions within the core possess a large disparity of temperatures, with some reaching above 37.7 °*C*. This is due to the limited distribution of blood caused by the low number of branch terminations associated with the vessel trees. This issue is averted with the flow reversal constant and larger vessel trees as the blood is distributed quickly throughout the entire tissue domain.

**Figure 5:**
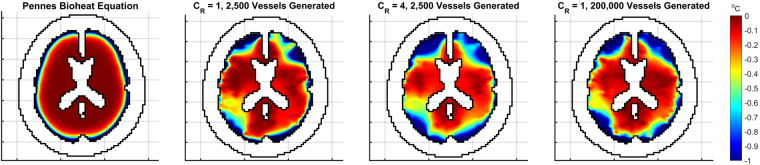
Profiles of tissue temperature difference when the scalp temperature is reduced from 33.5 °*C* to 10 °*C* in an adult head. The scale shows temperature differences from 0 °*C* to −1 °*C* for clarity.

Figure 5 shows the differences in temperature when scalp temperature is reduced from 33.5 °*C* to 10 °*C*. All cases using the VaPor model show greater cooling, with average brain temperature reductions from 0.56 °*C* (±<0.01 °*C*) to 0.58 °*C* (±<0.01 °*C*) depending on degree of vessel generation or value of *C*_*R*_, as compared to the temperature drop from PBE alone, 0.39 °*C*. Additionally, with increased counter-current flow of *C*_*R*_ = 4 and with increased vessel generation, the core regions of the brain (core regions being defined as voxels that possess >50% white matter in the input tissue fractions) experience larger drops in temperatures on average, from 0.29 °*C* (±<0.01 °*C*) to 0.45 °*C* (±<0.01 °*C*), compared to PBE, 0.11 °*C*. Here, the counter-current flow acts to effectively increase the thermal diffusivity of the tissue. This dampens the sharp temperature incline seen at the surface in the PBE model. A similar effect is seen with the tortuosity of the created vessels, where a similar amount of thermal mixing is produced leading to an increase of cooling in core regions. These results agree with the magnitude of cooling observed in Harris *et al*.^19^ where head cooling produced an average temperature reduction of 0.45 °*C*.

**Figure 6:**
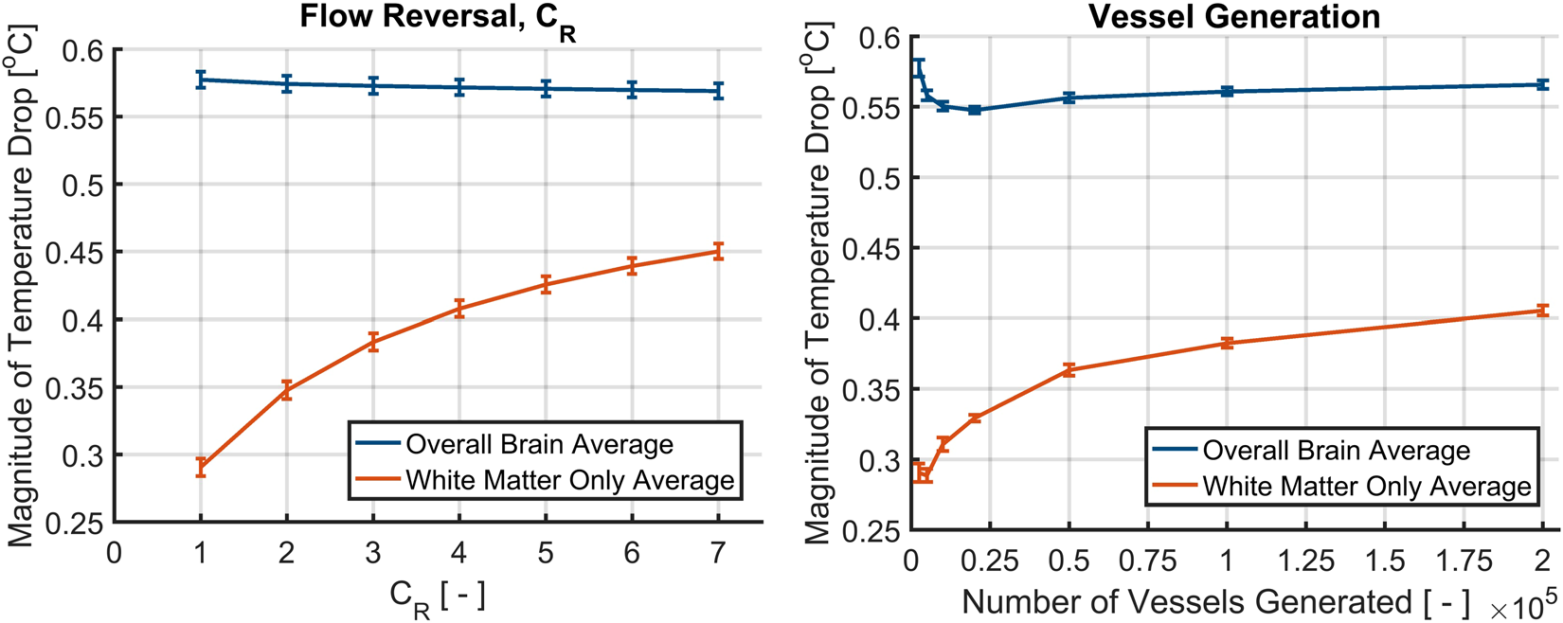
Graphical representation of average tissue temperature drop when scalp temperature is reduced from 33.5 °*C* to 10 °*C* in an adult head for various values of *C*_*R*_ and number of vessels generated.

Figure 6 shows graphically that increasing *C*_*R*_ shows increased cooling of the core regions, here expressed as tissue that contained >50% white matter in the input data. However, the overall drop in brain temperature remains relatively constant. A similar trend is observed when the number of generated vessels is increased within the model. The magnitude of average cooling with 200,000 vessels generated corresponds with *C*_*R*_ = 4 with only 2,500 vessels generated. This demonstrates the enhanced thermal exchange that occurs with the counter-current or tortuous flows associated with each method.

### Cooling a Neonatal Brain

**Figure 7:**
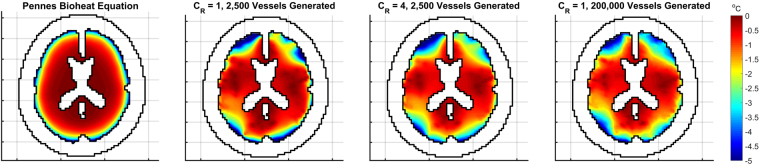
Profiles of tissue temperature difference when the scalp temperature is reduced from 33.5 °*C* to 10 °*C* in a neonatal scale head. The scale shows temperature differences from 0 °*C* to −5 °*C* for clarity.

The same trials are performed on a reduced scale model to replicate previous work on modelling neonatal heads under cooling conditions^18^. The domain was scaled by a factor of 11/15, the perfusion is reduced so that the average is 30 *ml*/100 *g*/*min*, which reduces the total inlet flow rate to 3.1 *g*/*s*, and the metabolism is reduced by 50%. All other parameters remain the same as the adult brain. Figure 7 shows the tissue temperature differences for a neonatal head with scalp temperature reduced from 33.5 °*C* to 10 °*C*.

The results for adult-sized brains demonstrate a small but significant reduction of core brain temperature not seen with PBE. However, with a neonatal brain, a much more pronounced level of cooling is observed. With *C*_*R*_ = 4, average cerebral tissue temperature is reduced to 35.23 °*C* (±0.02 °*C*, all errors denote the standard error across the 10 different vascular trees used) with the core regions being reduced to 35.70 °*C* (±0.02 °*C*). Similarly, with 200,000 vessels generated, cerebral tissue temperature is reduced to 35.15 °*C* (±0.02 °*C*) with the core regions being reduced to 35.49 °*C* (±0.02 °*C*). In contrast, with the PBE model, although the average temperature drops to 35.72 °*C*, the core region only falls to 36.47 °*C* which is above the mild hypothermic threshold of 36 °*C*.

## Discussion

We conducted simulations to investigate the impact of counter-current heat transfer in cerebral tissue using a vascular porous (VaPor) model. Initially, the counter-current flow of small vessels was simulated using a flow reversal constant *C*_*R*_ within the porous media domain. Subsequently, this was replicated by generating vessels from the initial vessel tree. Additional heat transfer to the core regions of the brain are seen in both cases with the flow-reversal constant and increased numbers of vessels. Hypothermic temperatures were produced in core regions for the case of a neonatal brain in contrast to previous studies of the same.

The VaPor model predicts human brain temperatures that agree with previous models under normal conditions and with observed temperatures after cooling from Harris *et al*.^19^. These results are difficult to validate due to the lack of *in vivo* measurements of human brain temperature for direct comparison. Previous validation for bioheat models is generally performed on smaller scales such as a bovine tongue^17^ or rat brains^10^. However, good agreement had previously been observed between the VaPor model and the ‘temperature shielding effect’ model for vertical temperature profiles in rat-scale brain simulations^31^.

With MRI thermometry, spatial thermal heterogeneity is observed^5^ which agrees with the temperature profiles produced with the VaPor model. With only the base vessel tree, without the flow-reversal constant, inter-domain blood transfer is limited to small regions which results in larger flow rates. This is due to the higher flow rate of blood in perfused regions limiting the temperature increase due to the energy released by metabolism which agrees with observations made using PBE^32^. In contrast, regions further away from these transfer points have lower flow rate and are therefore much more sensitive to variations in scalp temperature. With increased numbers of vessels, the distribution of flow corresponds to the expected metabolism resulting in less variation of temperatures. When using the flow-reversal constant, there exists higher thermal diffusivity which dampens out these regions of high temperature.

Upon applying a lower temperature to the scalp, it can be seen that the VaPor model predicts a larger degree of core cooling in the instances with increased thermal diffusivity. The magnitude of cooling observed in these cases corresponds to measurements obtained from thermal MRI scans^19^. This contradicts previous models that claim core cerebral temperatures cannot be affected by scalp cooling alone. However, within those trials, only PBE (or PBE combined with a limited number of vessels) is used to represent the convective heat transfer of blood flow. Because of this, counter-current effects from flow are not included. It is expected that similar cooling would be observed if the same level of vessel generation is employed in the DiVa model.

Although the small vessels rapidly reach equilibrium with the surrounding tissue as Chato suggests, the directional flow of these vessels is important too and therefore should not be omitted in calculations. This has been suggested before in previous bioheat models^14,33^, however, this only focussed on counter current heat exchange of major vessels. Here it can be seen that counter-current flow is important at all scales of blood flow.

Further cooling temperatures can be expected if the cooled blood from the brain is recirculated throughout the cardiovascular system, reducing the inlet arterial blood temperature. This has been demonstrated in other models^34^ but is not implemented here. The recirculation effect is expected to be more pronounced with the larger degree of cooling demonstrated with the VaPor model. This effect can be avoided in practice through the use of warming blankets.

Similarly, additional cooling is expected if the metabolic heat generation of tissue, *Q*_*gen*_, is considered a function of tissue temperature, *T*_2_, as implemented in previous models^34,35^. This relationship was omitted in the present study primarily for simplicity as it is non-linear, meaning the solutions could not be obtained through matrix inversion. Converting the solver to an explicit method drastically increases the calculation time, rendering solutions at the current resolution infeasible with available computing power. However, the impact of this omission is expected to be small. Michenfelder and Milde demonstrated in canine brains that on a reduction of temperature by 10 °*C*, the metabolic rate decreased by a factor of 3^36^. Using this correlation, the overall rate of cerebral metabolism in an adult brain would be reduced by 6% using the VaPor model compared to 4% with PBE. Metabolic heat generation only increases temperature by 0.24–0.27 °*C* at normal scalp temperature which means the impact from this reduction would only be of the order of 0.01 °*C*.

The original arterial and venous structures obtained omit any vasculature for the cerebellum (Fig. 2c), however this region is present in the brain data used. Although this is filled when expanding the vessel trees (Fig. 2d), the feeding arteries are not located correctly. As the algorithm joins generated points to the closest segment, points generated inside the cerebellum connect to the posterior branches of the middle cerebral arteries rather than the basilar artery. This alters the path taken by blood to perfuse this region and could have an impact on temperature. However, as the relative size of the cerebellum to the rest of the brain is low, the error caused by this is assumed to be low.

Due to the lack of vessel information for the head tissue surrounding the brain, the blood perfusion in these regions was simplified to the PBE model (Equation 1) in the current trials. However, there would exist some convective effects associated with the vasculature present here too, possibly increasing the cooling of tissue further. The circulation of cerebrospinal fluid flow surrounding the brain could also impact thermal transfer and help to maintain or cool the brain. More information is needed to implement these factors into the model.

The correct value for the flow reversal constant, *C*_*R*_, is uncertain as it is dependent on local vasculature. Here, it is considered to be homogeneous but in reality it would depend on local vasculature. This limitation is seen in previous bioheat models (such as the Chen and Holmes^14^ or Weinbaum and Jiji^33^ models) when approximating continuum variables for vascular properties and because of this, many studies revert back to PBE because of its simplicity. It was shown here that a value *C*_*R*_ = 4 yields results that correspond to the highest level of vessel generation available (200,000 iterations). Furthermore, it can be seen in Fig. 6 (right) that the change of temperature with increased vasculature appears to be plateauing, suggesting that further increasing vascular detail would yield little effect on tissue temperatures. Therefore the value *C*_*R*_ = 4 is a good estimate for the cerebral vasculature generation used within this study. For future applications, it is expected that local values for *C*_*R*_ could be experimentally derived from vasculature scans.

The VaPor model shows increased cerebral cooling when reducing the scalp temperature as compared to previous models using PBE. It also shows an increase in core brain cooling and less of a sharp slope in temperature profile within the surface tissue. The model depicts the blood vessels encouraging mixing between neighboring voxels which allows the overall tissue temperature to become more uniform. This has clinical potential for a cooling intervention by potentially offsetting damage inflicted by elevated tissue temperatures.

Target tissue temperature of <36 °*C* for brain cooling was met within neonatal models when the scalp temperature was reduced to 10 °*C*. If realised, this could be dramatically beneficial for reducing the long term damage from complications arising at birth without the need for inducing full body cooling, where systemic (core) temperature could be maintained <36°*C* while cooling the brain. Although none of the models with the adult brain reach the threshold of hypothermic tissue temperatures (<36 °*C*), the core reduction of 0.5 °*C* in core tissue could still provide medical benefit.

The presented VaPor model lends itself to the potential investigation of thermal intervention in events such as head trauma, for instance via cooling cap. Furthermore, the applicability of this model extends further than just thermal transport depicted here. Due to the vascular nature of the model, alterations to the model are simple, for instance, simulating an obstruction within a vessel in the event of ischemic stroke. This can be combined with modelling the transport of an arbitrary scalar quantity within blood, using a similar method as above, to emulate drug or contrast agent distribution in such an occurrence. The flexibility of the VaPor model lends itself to a wide range of clinical applications.

## Electronic supplementary material

LaTeX Supplementary File

## References

[1] Arrich, J., Holzer, M., Havel, C., Müllner, M. & Herkner, H. Hypothermia for neuroprotection in adults after cardiopulmonary resuscitation. *The Cochrane Libr*.,**2** (2016). 10.1002/14651858.CD004128.pub4 PMC6516972 26878327

[2] Crossley S (2014). A systematic review of therapeutic hypothermia for adult patients following traumatic brain injury. Critical Care.

[3] Saigal S, Sharma JP, Dhurwe R, Kumar S, Gurjar M (2015). Targeted temperature management: current evidence and practices in critical care. Indian J. Critical Care Medicine.

[4] Mariak Z (2002). Intracranial temperature recordings in human subjects the contribution of the neurosurgeon to thermal physiology. J. Therm. Biol..

[5] Thrippleton MJ (2014). Reliability of mrsi brain temperature mapping at 1.5 and 3 t. NMR Biomed..

[6] Pennes HH (1948). Analysis of tissue and arterial blood temperatures in the resting human forearm. J. Appl. Physiol.

[7] Nelson D, Nunneley S (1998). Brain temperature and limits on transcranial cooling in humans: quantitative modeling results. Eur. J. Appl. Physiol. Occup. Physiol..

[8] Zhu L, Diao C (2001). Theoretical simulation of temperature distribution in the brain during mild hypothermia treatment for brain injury. Med.Biol. Eng.Comput..

[9] Sukstanskii A, Yablonskiy D (2004). An analytical model of temperature regulation in human head. J. Therm. Biol.

[10] Zhu M, Ackerman JJ, Sukstanskii AL, Yablonskiy DA (2006). How the body controls brain temperature: the temperature shielding effect of cerebral blood flow. J. Appl. Physiol..

[11] Zhu M, Ackerman JJ, Yablonskiy DA (2009). Body and brain temperature coupling: the critical role of cerebral blood flow. J. Comp. Physiol B.

[12] Wulff, W. The energy conservation equation for living tissue. *IEEE Transactions on Biomed. Eng*. 494–495 (1974).

[13] Klinger H (1978). Heat transfer in perfused biological tissue-ii. the ‘macroscopic’ temperature distribution in tissue. Bull. Math. Biol..

[14] Chen MM, Holmes KR (1980). Microvascular contributions in tissue heat transfer. Annals New York Acad. Sci..

[15] Kotte A (1996). A description of discrete vessel segments in thermal modelling of tissues. Phys. Medicine Biol.

[16] Kotte A, Van Leeuwen G, Lagendijk J (1999). Modelling the thermal impact of a discrete vessel tree. Phys. Medicine Biol..

[17] Raaymakers B, Crezee J, Lagendijk J (2000). Modelling individual temperature profiles from an isolated perfused bovine tongue. Phys. Medicine Biol..

[18] Van Leeuwen GM, Hand JW, Lagendijk JJ, Azzopardi DV, Edwards AD (2000). Numerical modeling of temperature distributions within the neonatal head. Pediatr. Res..

[19] Harris B, Andrews P, Marshall I, Robinson T, Murray G (2008). Forced convective head cooling device reduces human cross-sectional brain temperature measured by magnetic resonance: a non-randomized healthy volunteer pilot study. Br. J. Anaesth..

[20] Chato J (1980). Heat transfer to blood vessels. ASME J. Biomech. Eng..

[21] Bergman, T. L., Incropera, F. P., DeWitt, D. P. & Lavine, A. S. *Fundamentals of heat and mass transfer* (John Wiley & Sons 2011).

[22] Kolios M, Sherar M, Hunt J (1995). Large blood vessel cooling in heated tissues: a numerical study. Phys. Medicine Biol.

[23] Krejza J (2006). Carotid artery diameter in men and women and the relation to body and neck size. Stroke.

[24] Hoskins, P. R. Chapter 8: The microcirculation. In *Cardiovascular Biomechanics*, 144 (Springer 2017).

[25] Reichold J (2009). Vascular graph model to simulate the cerebral blood flow in realistic vascular networks. J. Cereb. Blood Flow & Metab..

[26] Bullitt E, Gerig G, Pizer SM, Lin W, Aylward SR (2003). Measuring tortuosity of the intracerebral vasculature from mra images. IEEE transactions on medical imaging.

[27] Wright SN (2013). Digital reconstruction and morphometric analysis of human brain arterial vasculature from magnetic resonance angiography. Neuroimage.

[28] LaValle SM, Kuffner JJ (2001). Randomized kinodynamic planning. The Int. J. Robotics Res..

[29] Tanaka H (2006). Relationship between variations in the circle of willis and flow rates in internal carotid and basilar arteries determined by means of magnetic resonance imaging with semiautomated lumen segmentation: reference data from 125 healthy volunteers. Am. J. Neuroradiol.

[30] Craciunescu OI, Clegg ST (2001). Pulsatile blood flow effects on temperature distribution and heat transfer in rigid vessels. J. biomechanical engineering.

[31] Blowers, S. Modelling Brain Temperatures in Healthy Patients and Those with Induced Hypothermia. Ph.D. thesis, University of Edinburgh (2018).

[32] Sukstanskii AL, Yablonskiy DA (2006). Theoretical model of temperature regulation in the brain during changes in functional activity. Proc. Natl. Acad. Sci..

[33] Weinbaum S, Jiji L (1985). A new simplified bioheat equation for the effect of blood flow on local average tissue temperature. ASME J. Biomech. Eng..

[34] Neimark MA, Konstas A-A, Laine AF, Pile-Spellman J (2007). Integration of jugular venous return and circle of willis in a theoretical human model of selective brain cooling. J. Appl. Physiol..

[35] Neimark MA, Konstas A-A, Choi JH, Laine AF, Pile-Spellman J (2008). Brain cooling maintenance with cooling cap following induction with intracarotid cold saline infusion: a quantitative model. J. Theor. Biol..

[36] Michenfelder JD, Milde JH, Katušić ZS (1991). Postischemic canine cerebral blood flow is coupled to cerebral metabolic rate. J. Cereb. Blood Flow & Metab..

[37] Larsson HB, Courivaud F, Rostrup E, Hansen AE (2009). Measurement of brain perfusion, blood volume, and blood-brain barrier permeability, using dynamic contrast-enhanced t1-weighted mri at 3 tesla. Magn. Reson. Medicine.

